# Who values competent minds and who likes warm hearts? The role of right‐wing authoritarianism and social dominance orientation in shaping voter preferences for political candidates

**DOI:** 10.1111/bjso.70004

**Published:** 2025-07-06

**Authors:** Feiteng Long, Zi Ye

**Affiliations:** ^1^ Social, Economic and Organisational Psychology Leiden University Leiden The Netherlands

**Keywords:** competence, RWA, SDO, voting preferences, warmth

## Abstract

Decades of research have recognized political candidates' competence and warmth as predictors of voter preferences, but to whom these distinct personalities are most appealing remains unclear. In the current research, we investigated how voters' Right‐Wing Authoritarianism (RWA) and Social Dominance Orientation (SDO) influenced their preferences for highly competent or warm political candidates. In two studies, we experimentally manipulated candidate competence (Study 1) and warmth (Study 2) and measured voter preferences using overall evaluations, the feeling thermometer, and the likelihood‐of‐voting rating. We also measured perceived ability to clean up danger, perceived ability to win competitions, and perceived caring about people as potential mediators. The results suggested that a candidate's high competence (vs. neutral traits) increased preferences for the candidate among voters high in RWA more than those low in RWA. However, despite some indication, the effect that a candidate's high warmth (vs. neutral traits) increased such preferences among voters low in RWA more than those high in RWA lacked robustness across different voting preference measures. Additionally, the moderating effects of SDO on the relationships between candidate traits and voter preferences were not significant. Neither RWA nor SDO moderated the indirect effects of candidate competence and warmth through the proposed mediators.

## INTRODUCTION

Elections, which offer choices for voters between alternative political candidates, are at the very heart of the modern democratic system. Regardless of the institutional‐ and partisan‐level specificities, one of the most obvious cues guiding voters' electoral decisions is individual candidates' personalities. Decades of research in political science and social psychology have found that political candidates' success in elections partially depends on voters' positive perceptions of candidate personalities (e.g. Bishin et al., 2006; Catellani & Alberici, 2012; Fridkin & Kenney, 2011; Funk, 1996; Kinder et al., 1980; Laustsen, 2017; Miller et al., 1986). Among these personalities, competence and warmth are two fundamental dimensions of voters' social judgement of political candidates.

Over the past decades, a large body of research has examined the influences of candidate competence and warmth on voting preferences. Classic political science literature has long contended that competence is a key dimension of candidate personality based on which voters make their electoral decisions—they typically prefer competent candidates who promise strong leadership (Funk, 1996, 1997, 1999; McGraw, 2011). Social psychological research and recent political scientific studies, however, suggest that warmth is more fundamental than competence in people's judgements of social others (Da Silva & Costa, 2019; Fiske, 2018; Fiske et al., 2007; Laustsen & Bor, 2017). In fact, these seemingly contradictory findings may be explained by individual differences, particularly voters' ideological beliefs, that condition the respective impacts of candidate competence and warmth on voting preferences.

In the current research, we aimed to clarify ambiguities regarding the ideological differences in the influence of candidate competence/warmth on voting preferences. Two relevant ideological variables are Right‐Wing Authoritarianism (RWA) and Social Dominance Orientation (SDO) since they may reflect varied preferences for different patterns of leadership through people's dangerous and competitive (versus safe and cooperative) worldviews. Across two experimental studies conducted in the British political context, we tested the hypotheses that high candidate competence would increase voting preferences particularly among voters high in RWA and SDO, while high warmth would do so mainly among voters with low levels of RWA and SDO. To further drill down into the psychological mechanisms that might drive these effects, we proposed three potential mediators, namely the candidate's perceived ability to clean up danger, perceived ability to win competitions, and perceived caring about people.

### Voters' preferences for competent and warm candidates

In the past few years, social psychological research has established that competence and warmth are the two universal dimensions of people's judgement of social targets. According to social judgement theories and research, competence captures traits such as intelligence, capability and assertiveness, whereas warmth reflects sociability, friendliness, morality and trustworthiness (Cuddy et al., 2009; Fiske, 2015, 2018; Fiske et al., 2002, 2007). These dimensions also implicate one's social structural characteristics, especially status and competition—people of high social status (e.g. economically successful or well‐educated) are usually deemed to be competent, while competitors are perceived as threatening and consequently lacking warmth (Durante et al., 2013; Fiske et al., 1999, 2002, 2016). According to the stereotype content model (SCM; Cuddy et al., 2009; Fiske, 2018), perceptions of others as being competent and warm usually lead to positive emotions towards them, such as admiration and pride, while perceptions of lacking competence and warmth elicit greater negativity, like contempt and disgust. As a result, people tend to respect competent others and like those who are perceived as warm (Fiske et al., 1999, 2007). Recent developments based on the bi‐dimensional social judgement model have broken the warmth dimension down into sociability and morality (Brambilla et al., 2011; Brambilla & Leach, 2014). As Brambilla and colleagues contend, traits related to sociability and morality are differently processed and thus predict different judgement strategies when forming impressions and making inferences about others (Brambilla et al., 2011). Likewise, the similar rationale underlies competence that has been disentangled as ability and assertiveness (Abele, 2022; Abele et al., 2008, 2016; Carrier et al., 2014)—the former can be understood as intelligence and skills, whereas the latter stands for ambition and self‐confidence. In the current research, we focus on the higher‐level constructs of competence and warmth while also introducing their subcomponents to operationalize these traits more precisely.

In decades of electoral research, considerable cross‐sectional and experimental evidence has supported the positive role of competent and warm personalities of a political candidate in shaping candidate evaluations and voting preferences (e.g. Bishin et al., 2006; Catellani & Alberici, 2012; Fridkin & Kenney, 2011; Funk, 1996; Kinder et al., 1980; Laustsen, 2017; Miller et al., 1986). These two facets of social judgement, however, do not equivalently predict attitudes and behavioural tendencies towards the candidate. On the one hand, classic electoral research typically supports the argument that perceptions of competence more strongly predict preferences for specific political candidates than perceptions of warmth (Funk, 1996, 1997, 1999; McGraw, 2011). For example, as evidence from multiple elections shows, people tend to vote for political candidates who are perceived as both more competent and warmer, but only competence judgements consistently predict voting preferences across different elections (Funk, 1999). As one of the major responsibilities of a political leader is to handle the most urgent problems and shared goals of a group of people, which usually demand high intelligence, knowledge, and skills, voters should be sensitive to candidates' competence to ensure the realization of their collective goals (Funk, 1997; Popkin, 1994). Ironically, some works even reveal a “backfire” effect of warmth, namely that voters are less likely to support and trust candidates who are perceived as warmer under certain conditions (Castelli et al., 2009; Tan & Kraus, 2018). On the other hand, warmth appears to predict positive social judgements and be more influential than competence according to recent theories and evidence (e.g. Da Silva & Costa, 2019; Fiske, 2018; Fiske et al., 2007; Laustsen & Bor, 2017). As social psychologists argue, warmth reflects other people's apparent intent for good or ill, whereas competence implies the ability to enact this intent (Fiske, 2018). From an evolutionary perspective, as a social other's (good or ill) intent is pivotal to the survival of oneself, the other person's warmth‐related traits are processed before competence‐related ones in social judgements and carry a primary role in affective and behavioural reactions to the other person (Fiske et al., 2007). Recent evidence from American National Election Studies and an original experiment supports this prediction and reveals that candidate warmth is more influential to voters' affective reactions and voting preferences than other candidate personalities such as competence, leadership, and integrity (Laustsen & Bor, 2017). Although these findings offer initial evidence that both competence and warmth of a political candidate are desirable, there remains ambiguity as to individual differences—who particularly desires competence and who particularly favours warmth?

### RWA, SDO and voters' preferences for competent and warm candidates

Recent work has tried to clarify the inconsistent predictions about the roles of candidate competence and warmth in voters' preferences by examining voters' individual differences. For example, Laustsen (2017) found that conservative people prefer powerful candidates whereas liberals favour warm ones. According to their explanation, this ideological difference in candidate preferences depends on the way that people view the world (Laustsen, 2017). Specifically, conservatives tend to view the world as dangerous and competitive, such that they favour powerful political candidates who are competent enough to tackle the threats and uncertainties in the dangerous and competitive world. In contrast, liberals are more likely to perceive the world as safe and cooperative and therefore prefer warm candidates. However, as already established in the social psychology literature, RWA and SDO, instead of the liberal‐conservative political orientation, are the ideological beliefs that are most directly associated with dangerous/safe and competitive/cooperative worldviews, respectively (Duckitt, 2006; Duckitt et al., 2002; Duckitt & Sibley, 2009; Perry et al., 2013; Sibley et al., 2007). Most recent social psychological research even suggests that the belief in a dangerous world and the perception of the world as cooperative only explain a very small portion of variance in the liberal‐conservative political orientation (Clifton & Kerry, 2023). While Laustsen and Petersen's (2017) work provides initial evidence that people with high SDO (but not RWA) are attracted by the dominant faces of political candidates, the way that competence, a fundamental social judgement dimension that is related to but conceptually different from dominance (Laustsen & Petersen, 2018; Long et al., 2024), may interact with RWA and SDO remains unclear.

To address this question, we investigated the moderating roles of RWA and SDO, as two of the most relevant ideological variables, in the relationships between candidate personalities and voters' preferences. As individuals high in RWA perceive the world as dangerous and are sensitive to threats from the outside world (Cohrs & Asbrock, 2009; Dallago et al., 2012), they (compared to those relatively low in RWA) desire a highly competent political leader who can offer a sense of security and protect them from threats (Hypothesis 1a). In a similar vein, as those high in SDO tend to believe that zero‐sum competitions are ubiquitous, they admire top dogs while disparaging underdogs (Does & Mentovich, 2016; Ho et al., 2012; Hoyt & Simon, 2016). Hence, those social dominators (compared to those with a relatively low SDO) also prefer competent political candidates who can lead them to prevail in ruthless competitions (Hypothesis 1b). In contrast, we predict individuals low in RWA (Hypothesis 2a) and SDO (Hypothesis 2b) to particularly favour warm candidates since warmth is among the most preferred personalities in a peaceful and cooperative society experiencing fewer external threats. In testing these predictions, our work contributes to the literature on both political science and social psychology, addressing the present ambiguities and complexities of ideological differences in voting preferences for competent and warm candidates.

### Mechanisms underlying the influences of RWA and SDO

As already mentioned above, individuals with a high level of RWA view the world as a potentially dangerous place and are particularly sensitive to external threats (Cohrs & Asbrock, 2009; Dallago et al., 2012). Therefore, those high in RWA tend to prefer highly competent political leaders, as such leaders provide a reassuring sense of security and the promise of protection in a dangerous world. Likewise, social dominators, who view life as a series of zero‐sum competitions (Does & Mentovich, 2016; Ho et al., 2012; Hoyt & Simon, 2016), are drawn to competent leaders because they are seen as better equipped to navigate and succeed in a competitive, often unforgiving environment.

On the contrary, people low in RWA and SDO view the world as safer and more cooperative (Duckitt, 2006; Duckitt et al., 2002; Duckitt & Sibley, 2009; Perry et al., 2013; Sibley et al., 2007). In a secure and pro‐cooperation environment, people may prioritize the establishment of reciprocal relations rather than coping with environmental threats. Warmth, which signals one's good intent to build reciprocal relations (Cottrell & Neuberg, 2005; Eisenbruch & Krasnow, 2022), is particularly valued in this context. Hence, warm leaders tend to be more appealing to individuals low in RWA and SDO, as they are typically perceived as caring for the general public—reflecting a form of reciprocity relationship between politicians and voters).

Based on this line of reasoning, we predict that the indirect effects of candidate competence on voting preferences through the candidate's perceived ability to clean up danger and perceived ability to win competitions will be moderated by RWA (Hypothesis 3) and SDO (Hypothesis 4), respectively. Specifically, these indirect effects are expected to be stronger among individuals with higher levels of RWA and SDO. Likewise, the indirect effect of candidate warmth on voting preferences through the candidate's perceived caring about people will also be moderated by RWA (Hypothesis 5a) and SDO (Hypothesis 5b). This indirect effect is anticipated to be more pronounced among individuals lower in RWA and SDO.

### Overview of the present research

In the current research, we examined how voters' RWA and SDO moderate the influences of candidate competence and warmth on voting preferences, along with the underlying mechanisms. Specifically, we predicted that a candidate's high competence would enhance voters' preferences for them, particularly among voters high in RWA (Hypothesis 1a) and SDO (Hypothesis 1b). This is because a highly competent candidate is perceived as capable of cleaning up danger and winning competitions (leading to Hypotheses 3 and 4). On the other hand, a candidate's high warmth would increase voters' preferences for them, particularly among voters low in RWA (Hypothesis 2a) and SDO (Hypothesis 2b), as warm candidates are perceived as caring about people (leading to Hypotheses 5a and 5b). To test these predictions, we conducted two studies with British participants. In Study 1, we manipulated competence by contrasting a high‐competence condition with a neutral condition, while in Study 2, we manipulated warmth by contrasting a high‐warmth condition with a neutral one. The inclusion of a neutral (baseline) condition allowed us to control for the possibility that individuals higher in RWA and SDO may report a general preference for any political candidate, regardless of their traits. This design enabled us to determine whether the observed effects were uniquely driven by competence or warmth, rather than reflecting a broad pro‐leader bias among high‐RWA and high‐SDO individuals.

The present research is a Stage 2 Registered Report, with the accepted Stage 1 protocol preregistered at https://osf.io/zaufb. The materials and data used in the current studies are accessible at https://osf.io/x3dp7. The studies were approved by the Psychology Research Ethics Committee at Leiden University, and informed consent was obtained from participants.

## STUDY 1

Study 1 focused on candidate competence and aimed to test Hypotheses 1a and 1b regarding the moderating roles of RWA and SDO in the relationship between candidate competence and voting preferences. Additionally, we tested Hypotheses 3 and 4 regarding moderated mediations (i.e. the mediating roles of perceived ability to clean up danger and perceived ability to win competitions moderated by RWA and SDO).

### Methods

#### Participants

As little is known about the interactive effect of candidate personality and RWA/SDO on voting preferences in the existing literature, we based our power analysis on data that we collected as part of another project (Long et al., 2024), which suggests an effect size of $\eta_{p}^{2}$ = 0.04 (i.e. *f* ^2^ = 0.042) for the interaction between candidate personality (within‐participants: competence vs. warmth) and SDO (continuous measure). Since we used the PROCESS Model 15 (Hayes, 2013) to examine the moderated mediation, we calculated the sample size for a regression model with 10 predictors (i.e. a dummy variable for the manipulation, a moderator, an interaction term for manipulation × moderator, three mediators, and three interaction terms for mediator × moderator). The a priori power analysis on G*Power (Faul et al., 2007) suggested a sample size of 396 could yield a power of 0.80 to detect the anticipated effect size. Considering a possible exclusion rate of 15%, we planned to recruit 466 participants.

A total of 468 British nationals residing in the United Kingdom and eligible to vote were recruited via Prolific (www.prolific.com). Following data collection, 31 responses that failed one of the attention checks were excluded from our analyses. For the open‐ended attention check, participants who correctly answered either the first or last name of the political candidate were retained. The final sample comprised 437 valid responses. Participants were aged between 19 and 81 (M = 43.89, SD = 14.23). Most of them were women (278 women, 150 men, 4 non‐binary individuals, 4 females, and 1 transwoman), White (404 White participants, 16 Asian, 10 Black, and 7 mixed‐race), and well‐educated (252 degree holders and 185 non‐degree holders). Participants reported a mean socioeconomic status at the midpoint of the scale (M = 5.50, SD = 1.30) and were slightly left‐leaning in political orientation (M = 3.55, SD = 1.30).

#### Procedure

After providing informed consent, participants were randomly assigned to one of two conditions (candidate competence: high vs. neutral) in a between‐participants design. They then completed manipulation check items, followed by measures of research variables. The outcome measures were either presented before or after the mediation measures in a randomized order to balance the potential order effect, while the RWA and SDO scales were always presented after the outcome and mediation measures. At the end of the questionnaire, participants received a thorough debriefing.

#### Manipulation of candidate competence

**FIGURE 1:**
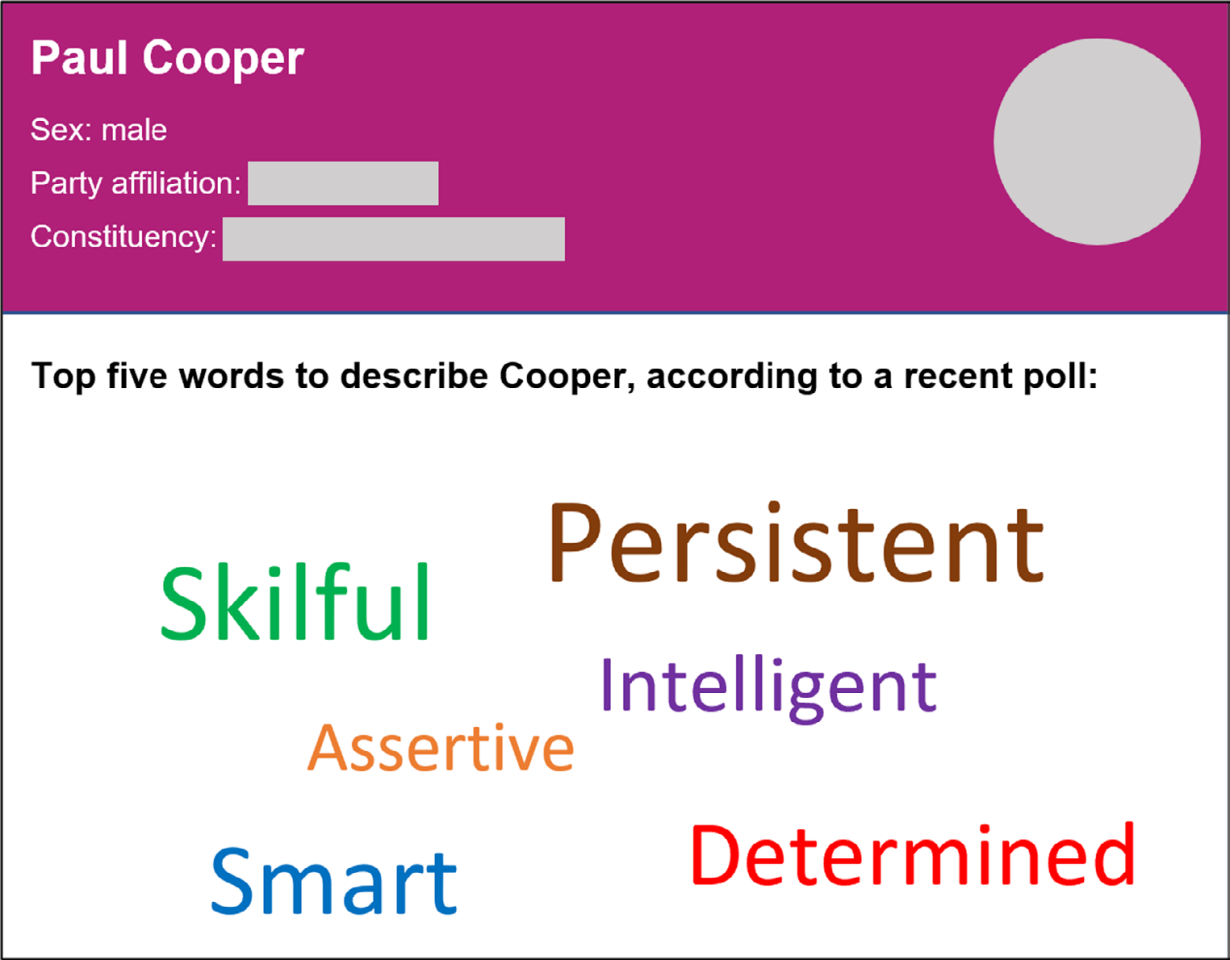
An example of the bogus profile along with the word cloud used in the manipulation.

We presented a profile of a male political candidate, Paul Cooper, who was said to be one of the most promising contenders for a seat in the House of Commons representing a certain constituency in the east of England. Below the profile, participants read a brief summary of a recent poll conducted among residents of the candidate's constituency, which aimed to survey public impressions of each candidate competing for the legislative seat. Following the paradigm used by Callaghan et al. (2022) and Long et al. (2024), participants were presented with a “word cloud” displaying Cooper's top five traits, ostensibly according to public opinion. Based on random assignment, participants learned that Cooper was described as either persistent, skilful, smart, determined, intelligent and assertive (high‐competence; Abele, 2022; Abele et al., 2016; Callaghan et al., 2022; Cambon, 2022; Cuddy et al., 2008); or objective, rational, practical, focused, unconventional and outspoken (neutral). An example of the contrived profile can be found in Figure 1.

Following the manipulation information, participants completed attention and manipulation checks. The attention check, presented as an open‐ended question, asked participants to recall the name of the candidate mentioned in the poll. The manipulation check was assessed on seven‐point scales anchored at 1 (*not at all*) and 7 (*very much*), asking participants to indicate the extent to which they considered Cooper to also have the following characteristics: capable, strong‐willed, competent and self‐confident (perceived candidate competence; *α* = .80).

#### Measures

As the outcome variable, voters' preferences for the candidate were operationalized in attitudinal (i.e. overall evaluations), affective (i.e. the feeling thermometer) and intentional (i.e. the likelihood of voting) terms. As potential mediators, we assessed perceived ability to clean up danger, perceived ability to win competitions, and perceived caring about people. RWA and SDO measures were administered subsequently, prior to demographic questions. Appendix S1 presents descriptive statistics and bivariate correlations between the measures, along with their means and standard deviations between experimental conditions.

##### Overall evaluations

Overall evaluations of the political candidate, as an operationalization of voting preferences, were assessed using three items: ‘Cooper is suitable for the position as an MP’; ‘Cooper will be a good politician’; ‘Supporting Cooper will be a wise choice for the voters in his constituency’. (1 = *strongly disagree* to 7 = *strongly agree*; *α* = .92).

##### Feeling thermometer

Overall feelings towards the political candidate were gauged using a feeling thermometer: ‘In general, how negative or positive do you feel towards Cooper?’ (0 = *very negative*, 50 = *neutral*, 100 = *very positive*).

##### Likelihood of voting

Participants were asked to indicate their likelihood of voting for Cooper, on a scale ranging from 0 to 100 (%).

##### Perceived ability to clean up danger

Participants were asked to what extent Cooper, if elected, would be able to contribute to: ‘prevent the country from getting more dangerous and chaotic’, ‘combat unpredictable threats to the society’, and ‘restore the security and order of the country’. (1 = *not at all* to 7 = *very much*; *α* = .92).

##### Perceived ability to win competitions

Likewise, participants indicated the extent to which that Cooper, if elected, would be able to: ‘survive in the political struggle’, ‘grab as many resources as possible to achieve his political goals’, and ‘defeat the opponents in the parliament’. (1 = *not at all* to 7 = *very much*; *α* = .77).

##### Perceived caring about people

Participants indicated the likelihood that, once elected, Cooper would: ‘care about the needs of people he represents’, ‘support policies in favour of people he represents’, and ‘seek more social services for people he represents’. (1 = *very unlikely* to 7 = *very likely*; *α* = .93).

##### RWA

Since making participants' RWA and SDO salient first may overstate the influence of these values on participants' subsequent evaluations, we presented the RWA and SDO measures after the outcome and mediation measures. We employed the RWA scale shortened by Bizumic and Duckitt (2018), which includes six items: ‘It's great that many young people today are prepared to defy authority’; ‘What our country needs most is discipline, with everyone following our leaders in unity’; ‘God's laws about abortion, pornography, and marriage must be strictly followed before it is too late’; ‘There is nothing wrong with premarital sexual intercourse’; ‘Our society does NOT need tougher government and stricter laws’; ‘The facts on crime and the recent public disorders show we have to crack down harder on troublemakers, if we are going preserve law and order’. (1 = *strongly oppose* to 7 = *strongly favour*; *α* = .78).

##### SDO

A short version of the SDO_7_ scale constructed by Ho et al. (2015) was employed to assess SDO. This scale includes eight items: ‘An ideal society requires some groups to be on top and others to be on the bottom’; ‘Some groups of people are simply inferior to other groups’; ‘No one group should dominate in society’; ‘Groups at the bottom are just as deserving as groups at the top’; ‘Group equality should not be our primary goal’; ‘It is unjust to try to make groups equal’; ‘We should do what we can to equalize conditions for different groups’; ‘We should work to give all groups an equal chance to succeed’. (1 = *strongly oppose* to 7 = *strongly favour*; *α* = .89).

##### Demographic questions

After presenting the RWA and SDO scales, we asked participants to provide information about age, gender (with choices ‘men’, ‘women’, ‘non‐binary’, and ‘I prefer to identify myself as…’), education attainment (with choices ‘primary education or lower’, ‘secondary education’, ‘vocational training’, ‘Bachelor's or equivalent degree’, ‘Master's or equivalent degree’, and ‘Doctoral or higher’), socioeconomic status (using the MacArthur Scale of Subjective Social Status; Adler et al., 2000), ethnic identity (with choices ‘Asian’, ‘Black’, ‘White’, and ‘I prefer to identify myself as…’) and political orientation (1 = *vey leftist* to 7 = *very rightist*).

##### Additional attention checks

In addition to the attention check following the manipulation information, we included two additional attention checks in the questionnaire. A simple attention check, ‘I would demonstrate my careful reading by selecting number six’, was embedded within the RWA and SDO scales. Another was presented among the demographic questions: ‘Which city is the capital of the United Kingdom?’ (1 = Amsterdam, 2 = Hong Kong, 3 = London, 4 = New York, 5 = Paris).

### Results

#### Factor analysis

As preregistered, we conducted factor analysis for the mediation scales, namely perceived ability to clean up danger, perceived ability to win competitions, and perceived caring about people. The results (see Table S2.1 in Appendix S2) show three components in line with our proposed three measures.

#### Manipulation check

An independent samples *t*‐test (1 = high‐competence, 0 = neutral) on the manipulation check showed no significant difference in perceived candidate competence between the high‐competence (*n* = 217, M = 5.78, SD = 0.75) and neutral (*n* = 220, M = 5.67, SD = 0.85) conditions, *t*(435) = 1.31, *p* = .190, *d* = 0.13. As preregistered, even though the manipulation did not successfully change participants' perceptions of candidate competence, it is still worthwhile to test the effect of making candidate competence salient through exposure.

#### Outcome variables

To test Hypotheses 1a and 1b, we employed the PROCESS Model 1 (Hayes, 2013), specifying the candidate competence manipulation as the independent variable (1 = high‐competence, 0 = neutral), RWA/SDO as the moderator, and each of the three voting preference measures (i.e. overall evaluations, the feeling thermometer and the likelihood of voting) as the dependent variable. We included RWA and SDO in separate models in order to fully account for their influences without controlling for each other. Means and standard errors of the outcome variables at different RWA/SDO levels can be found in Table 1.

**TABLE 1:** Means and standard errors for outcome variables in different experimental conditions and at different RWA/SDO levels in Study 1.

Outcome variable	Condition	Moderator: RWA		Moderator: SDO	
		M − SD	M + SD	M − SD	M + SD
Overall evaluations	High‐competence	4.74 (0.10)	5.53 (0.10)	4.97 (0.11)	5.32 (0.11)
	Neutral	4.86 (0.10)	5.24 (0.11)	4.80 (0.11)	5.28 (0.10)
Feeling thermometer	High‐competence	58.03 (1.62)	70.39 (1.60)	62.12 (1.66)	66.61 (1.70)
	Neutral	59.74 (1.63)	66.98 (1.66)	58.79 (1.67)	67.60 (1.63)
Likelihood of voting	High‐competence	51.79 (1.86)	66.96 (1.83)	55.79 (1.91)	63.40 (1.95)
	Neutral	54.75 (1.87)	62.35 (1.90)	53.91 (1.92)	62.86 (1.88)
Voting preference (composite)	High‐competence	−0.33 (0.09)	0.38 (0.08)	−0.12 (0.09)	0.19 (0.09)
	Neutral	−0.22 (0.09)	0.16 (0.09)	−0.26 (0.09)	0.19 (0.09)

*Note*: Entries are means with standard errors in parentheses.

In terms of the moderating role of RWA, Table 2 shows that the manipulation of candidate competence and RWA interacted in affecting overall evaluations and the likelihood of voting. Decomposition of the interactions revealed a marginal trend in which individuals high in RWA evaluated the highly competent political candidate more positively (*b* = 0.29, SE = 0.15, *p* = .051) and reported a higher likelihood of voting for him (b = 4.61, SE = 2.64, *p* = .082) compared to the neutral political candidate. However, among participants low in RWA, there was no significant difference in overall evaluations (*b* = −0.12, SE = 0.15, *p* = .411) and the likelihood of voting (*b* = −2.96, SE = 2.64, *p* = .263) between the two experimental conditions.

**TABLE 2:** The interactive effect of the manipulation of candidate competence and RWA/SDO on outcome variables in Study 1.

Predictor	Outcome variable			
	Overall evaluations	Feeling thermometer	Likelihood of voting	Voting preference (composite)
Moderator: RWA				
Manipulation	−0.52 (0.33), *p* = .109	−6.74 (5.09), *p* = .186	−10.39 (5.84), *p* = .076	−0.45 (0.27), *p* = .095
RWA	0.17 (0.07), *p* = .010	3.24 (1.06), *p* = .002	3.40 (1.21), *p* = .005	0.17 (0.06), *p* = .003
Manipulation × RWA	0.18 (0.09), *p* = .050	2.30 (1.46), *p* = .116	3.39 (1.68), *p* = .044	0.15 (0.08), *p* = .049
Moderator: SDO				
Manipulation	0.27 (0.27), *p* = .315	6.13 (4.18), *p* = .143	2.76 (4.81), *p* = .567	0.24 (0.22), *p* = .282
SDO	0.21 (0.06), *p* = .001	3.78 (1.00), *p* < .001	3.84 (1.15), *p* < .001	0.20 (0.05), *p* < .001
Manipulation × SDO	−0.06 (0.09), *p* = .499	−1.85 (1.43), *p* = .196	−0.58 (1.64), *p* = .726	−0.06 (0.08), *p* = .412

*Note*: Entries are unstandardized regression coefficients with standard errors in parentheses, followed by *p*‐values.

In addition, we conducted the above analyses using a composite score of voting preference as the dependent variable. This composite score was obtained by standardizing the three outcome measures (i.e. overall evaluations, the feeling thermometer, and the likelihood of voting) and averaging their *z*‐scores.^1^ Fitting a regression model, we found a significant interaction between candidate competence and RWA (see Table 2). Decomposition of the interaction revealed a marginal trend, with individuals high in RWA preferring the highly competent political candidate over the neutral one, *b* = 0.22, SE = 0.12, *p* = .066. However, among participants low in RWA, there was no significant difference in voting preference between the two experimental conditions, *b* = −0.12, SE = 0.12, *p* = .340. Altogether, these results suggest that a political candidate's high competence (vs. neutral traits) increased preferences for the candidate among voters high in RWA more than those low in RWA, thus supporting Hypothesis 1a. However, since the manipulation check was not successful, the manipulation effect on voting preferences was likely due to the exposure to salient competence‐related traits rather than perceived high competence.

Regarding SDO, Table 2 shows that the interaction between candidate competence and SDO on all three outcome variables was not significant. Furthermore, a parallel analysis using the composite voting preference measure as the dependent variable also revealed no significant interaction between candidate competence and SDO. These results, therefore, do not support Hypothesis 1b.

#### Moderated mediation

We tested Hypotheses 3 and 4 regarding moderated mediation using PROCESS Model 15 (Hayes, 2013), with candidate competence as the independent variable, RWA/SDO as the moderator, and each of the three voting preference measures as the dependent variable. In addition, perceived ability to clean up danger, perceived ability to win competitions, and perceived caring about people were specified as parallel mediators.

As shown in Table 3, the indirect effects of candidate competence on outcome variables through perceived ability to clean up danger were not moderated by RWA. A parallel analysis using the composite voting preference measure as the dependent variable also revealed no significant moderated mediation. These results do not support Hypothesis 3.

**TABLE 3:** Indirect effects of candidate competence moderated by RWA/SDO in Study 1.

Mediator	Moderator: RWA			Moderator: SDO		
	Estimate	SE	95% CI	Estimate	SE	95% CI
Indirect effect of candidate competence on overall evaluations						
Perceived ability to clean up danger	−0.01	0.01	[−0.03, 0.01]	0.01	0.01	[−0.01, 0.04]
Perceived ability to win competitions	0.01	0.02	[−0.02, 0.04]	0.02	0.01	[−0.01, 0.05]
Perceived caring about people	0.00	0.01	[−0.01, 0.03]	−0.00	0.01	[−0.02, 0.01]
Indirect effect of candidate competence on feeling thermometer						
Perceived ability to clean up danger	−0.07	0.13	[−0.36, 0.16]	0.11	0.17	[−0.18, 0.52]
Perceived ability to win competitions	0.11	0.20	[−0.28, 0.55]	0.31	0.20	[−0.04, 0.74]
Perceived caring about people	0.11	0.16	[−0.13, 0.50]	−0.00	0.14	[−0.30, 0.28]
Indirect effect of candidate competence on likelihood of voting						
Perceived ability to clean up danger	−0.07	0.15	[−0.42, 0.21]	0.13	0.21	[−0.23, 0.63]
Perceived ability to win competitions	−0.05	0.26	[−0.56, 0.52]	0.04	0.24	[−0.45, 0.53]
Perceived caring about people	0.01	0.21	[−0.44, 0.43]	−0.10	0.22	[−0.64, 0.26]
Indirect effect of candidate competence on voting preference (composite)						
Perceived ability to clean up danger	−0.00	0.01	[−0.02, 0.01]	0.01	0.01	[−0.01, 0.03]
Perceived ability to win competitions	0.00	0.01	[−0.02, 0.03]	0.01	0.01	[−0.01, 0.03]
Perceived caring about people	0.00	0.01	[−0.01, 0.02]	−0.00	0.01	[−0.02, 0.01]

*Note*: Confidence intervals (CIs) were obtained with 5000 bootstrap replicates.

Likewise, Table 3 shows that the indirect effects of candidate competence on outcome variables through perceived ability to win competitions were not moderated by SDO. A parallel analysis with the composite voting preference measure as the dependent variable again found no significant moderated mediation. These results do not support Hypothesis 4.

## STUDY 2

We conducted a parallel Study 2 to examine candidate warmth. In Study 2, we tested Hypotheses 2a and 2b regarding the moderating roles of RWA and SDO in the relationship between candidate warmth and voting preferences. Additionally, we aimed to test Hypotheses 5a and 5b regarding moderated mediations (i.e. the mediating role of perceived caring about people moderated by RWA and SDO).

### Methods

#### Participants

Based on the same rationale as in Study 1, we planned to recruit 466 participants for Study 2. A total of 465 British nationals residing in the United Kingdom and eligible to vote completed our study on Prolific (www.prolific.com). Following data collection, 21 responses that failed one of the attention checks were excluded from our analyses. For the open‐ended attention check, participants who correctly answered either the first or last name of the political candidate were retained. The final sample comprised 444 valid responses. Participants were aged between 18 and 81 (M = 46.09, SD = 14.00). Most of them were women (243 women, 199 men, 1 non‐binary individual, and 1 female), White (407 White participants, 15 Black, 12 Asian, 6 mixed‐race, 1 Brown, 1 Jewish, 1 Turkish Cypriot, and 1 unspecified) and well‐educated (253 degree holders and 191 non‐degree holders). Participants reported a mean socioeconomic status slightly below the midpoint of the scale (M = 5.32, SD = 1.49) and were slightly left‐leaning in political orientation (M = 3.64, SD = 1.24).

#### Procedure and materials

The procedure and materials were identical to those in Study 1, except for the manipulation. Specifically, we manipulated candidate warmth (i.e. high vs. neutral) instead of candidate competence in the current study. Following the same paradigm used in Study 1 (Callaghan et al., 2022; Long et al., 2024), participants learned that Cooper was described as either kind, honest, trustworthy, nice, sincere and friendly (high‐warmth; Abele, 2022; Brambilla et al., 2011; Bick et al., 2022; Callaghan et al., 2022); or objective, rational, practical, focused, unconventional and outspoken (neutral). Accordingly, the manipulation check assessed the extent to which participants considered Cooper to also have the following characteristics: sociable, reliable, good‐natured and genuine (perceived candidate warmth; *α* = .88).

The outcome, mediation, and moderation measures demonstrated good reliability (overall evaluations: *α* = .94; perceived ability to clean up danger: *α* = .93; perceived ability to win competitions: *α* = .78; perceived caring about people: *α* = .94; RWA: *α* = .74; SDO: *α* = .90). Again, descriptive statistics and bivariate correlations between the measures, as well as their means and standard deviations between experimental conditions, can be found in Appendix S1.

### Results

#### Factor analysis

Again, we conducted factor analysis for the mediation scales, namely perceived ability to clean up danger, perceived ability to win competitions and perceived caring about people. The analysis (see Table S2.2 in Appendix S2) indicated three components in line with our proposed three measures.

#### Manipulation check

An independent samples *t*‐test (1 = high‐warmth, 0 = neutral) on the manipulation check suggested that participants in the high‐warmth condition (*n* = 224, M = 5.67, SD = 0.88), compared to those in the neutral condition (*n* = 220, M = 4.64, SD = 1.01), reported higher perceptions of candidate warmth, *t*(442) = 11.37, *p* < .001, *d* = 1.08. Therefore, the manipulation of candidate warmth was successful.

#### Outcome variables

Regression analyses similar to those in Study 1 were conducted to test Hypotheses 2a and 2b. Means and standard errors of the outcome variables at different RWA/SDO levels can be found in Table 4.

**TABLE 4:** Means and standard errors for outcome variables in different experimental conditions and at different RWA/SDO levels in Study 2.

Outcome variable	Condition	Moderator: RWA		Moderator: SDO	
		M − SD	M + SD	M − SD	M + SD
Overall evaluations	High‐warmth	5.31 (0.11)	5.49 (0.11)	5.52 (0.11)	5.30 (0.11)
	Neutral	4.51 (0.10)	5.10 (0.11)	4.71 (0.11)	4.89 (0.11)
Feeling thermometer	High‐warmth	72.59 (1.78)	75.21 (1.73)	74.21 (1.79)	73.68 (1.73)
	Neutral	55.26 (1.69)	65.24 (1.74)	58.84 (1.75)	61.49 (1.81)
Likelihood of voting	High‐warmth	64.00 (2.12)	71.17 (2.06)	68.18 (2.15)	67.22 (2.07)
	Neutral	49.26 (2.02)	61.62 (2.07)	53.59 (2.09)	57.07 (2.17)
Voting preference (composite)	High‐warmth	0.19 (0.09)	0.38 (0.08)	0.33 (0.09)	0.25 (0.08)
	Neutral	−0.54 (0.08)	−0.03 (0.08)	−0.36 (0.08)	−0.22 (0.09)

*Note*: Entries are means with standard errors in parentheses.

Results reported in Table 5 reveal a significant interaction between candidate warmth and RWA on the feeling thermometer, suggesting that a candidate's high warmth (vs. neutral traits) increased positive feelings towards the candidate more among individuals low in RWA (*b* = 17.33, SE = 2.46, *p* < .001) than among those high in RWA (*b* = 9.96, SE = 2.46, *p* < .001). There was also a marginal interaction between candidate warmth and RWA on overall evaluations and composite voting preference. For exploratory purposes, we probed the simple slopes and found that individuals low in RWA (overall evaluations: *b* = 0.80, SE = 0.15, *p* < .001; voting preference: *b* = 0.73, SE = 0.12, *p* < .001) positively evaluated and preferred the highly warm candidate over the neutral candidate to a slightly greater extent than those high in RWA (overall evaluations: *b* = 0.39, SE = 0.15, *p* = .010; voting preference: *b* = 0.42, SE = 0.12, *p* < .001). These results, together, provide limited support for Hypothesis 2a given the lack of robustness across different voting preference measures.

**TABLE 5:** The interactive effect of the manipulation of candidate warmth and RWA/SDO on outcome variables in Study 2.

Predictor	Outcome variable			
	Overall evaluations	Feeling thermometer	Likelihood of voting	Voting preference (composite)
Moderator: RWA				
Manipulation	1.28 (0.38), *p* < .001	26.10 (6.13), *p* < .001	20.91 (7.30), *p* = .004	1.10 (0.30), *p* < .001
RWA	0.29 (0.07), *p* < .001	4.88 (1.17), *p* < .001	6.04 (1.39), *p* < .001	0.25 (0.06), *p* < .001
Manipulation × RWA	−0.20 (0.10), *p* = .060	−3.60 (1.70), *p* = .035	−2.54 (2.03), *p* = .211	−0.15 (0.08), *p* = .063
Moderator: SDO				
Manipulation	1.07 (0.27), *p* < .001	17.46 (4.46), *p* < .001	17.49 (5.35), *p* = .001	0.85 (0.22), *p* < .001
SDO	0.08 (0.07), *p* = .251	1.15 (1.09), *p* = .292	1.51 (1.31), *p* = .249	0.06 (0.05), *p* = .235
Manipulation × SDO	−0.17 (0.09), *p* = .068	−1.38 (1.54), *p* = .369	−1.93 (1.84), *p* = .297	−0.10 (0.07), *p* = .181

*Note*: Entries are unstandardized regression coefficients with standard errors in parentheses, followed by *p*‐values.

Regarding SDO, Table 5 indicates a marginally significant interaction between candidate warmth and SDO on overall evaluations. Exploratory decomposition of this interaction revealed that individuals low in SDO (*b* = 0.81, SE = 0.15, *p* < .001) evaluated the highly warm candidate more positively than the neutral candidate, to a slightly greater extent than those high in SDO (*b* = 0.41, SE = 0.15, *p* = .008). However, Table 5 demonstrates no significant interactive effect of candidate warmth and SDO on the feeling thermometer, the likelihood of voting, or composite voting preference. Therefore, we have very limited evidence in support of Hypothesis 2b.

#### Moderated mediation

We tested Hypotheses 5a and 5b regarding moderated mediation using PROCESS Model 15 (Hayes, 2013), with candidate warmth as the independent variable, RWA/SDO as the moderator, and each of the three voting preference measures as the dependent variable. In addition, perceived ability to clean up danger, perceived ability to win competitions, and perceived caring about people were specified as parallel mediators.

As shown in Table 6, the indirect effects of candidate warmth on outcome variables through perceived caring about people were not moderated by RWA or SDO. A parallel analysis using the composite voting preference measure as the dependent variable also revealed no significant moderated mediation. Hence, these results do not support Hypothesis 5a and 5b.

**TABLE 6:** Indirect effects of candidate warmth moderated by RWA/SDO in Study 2.

Mediator	Moderator: RWA			Moderator: SDO		
	Estimate	SE	95% CI	Estimate	SE	95% CI
Indirect effect of candidate warmth on overall evaluations						
Perceived ability to clean up danger	−0.00	0.01	[−0.01, 0.01]	0.00	0.01	[−0.01, 0.01]
Perceived ability to win competitions	0.00	0.03	[−0.05, 0.06]	0.01	0.03	[−0.03, 0.07]
Perceived caring about people	0.00	0.05	[−0.10, 0.09]	−0.01	0.04	[−0.10, 0.08]
Indirect effect of candidate warmth on feeling thermometer						
Perceived ability to clean up danger	−0.03	0.11	[−0.29, 0.17]	0.01	0.09	[−0.19, 0.19]
Perceived ability to win competitions	0.27	0.43	[−0.62, 10.08]	−0.38	0.41	[−10.14, 0.46]
Perceived caring about people	0.43	0.82	[−10.31, 10.94]	−0.06	0.78	[−10.67, 10.44]
Indirect effect of candidate warmth on likelihood of voting						
Perceived ability to clean up danger	−0.04	0.14	[−0.37, 0.22]	0.01	0.10	[−0.21, 0.21]
Perceived ability to win competitions	0.48	0.48	[−0.52, 10.37]	−0.49	0.45	[−10.37, 0.42]
Perceived caring about people	0.36	0.92	[−10.51, 20.05]	−0.65	0.85	[−20.46, 0.91]
Indirect effect of candidate warmth on voting preference (composite)						
Perceived ability to clean up danger	−0.00	0.00	[−0.01, 0.01]	0.00	0.00	[−0.01, 0.01]
Perceived ability to win competitions	0.01	0.02	[−0.03, 0.05]	−0.01	0.02	[−0.04, 0.03]
Perceived caring about people	0.01	0.03	[−0.06, 0.07]	−0.01	0.03	[−0.08, 0.05]

*Note*: Confidence intervals (CIs) were obtained with 5000 bootstrap replicates.

## DISCUSSION OF STUDIES 1 AND 2

In the current research, we investigated how voters' RWA and SDO influence their preferences for political candidates exhibiting high competence or warmth. Across two studies, we experimentally manipulated candidate competence (Study 1) and warmth (Study 2) and measured voting preferences using overall evaluations, the feeling thermometer, and the likelihood‐of‐voting rating. Overall, we predicted that high competence (vs. neutral traits) of the candidate would enhance voting preferences particularly among voters high in RWA and SDO, whereas high candidate warmth (vs. neutral traits) would play such a role particularly among voters low in RWA and SDO. Furthermore, we expected these influences to be mediated by the candidate's perceived ability to clean up danger, perceived ability to win competitions, and perceived caring about people.

Study 1 results suggested that a political candidate's high competence (vs. neutral traits) increased preferences for the candidate—except on the feeling thermometer—among voters high in RWA more than those low in RWA, supporting Hypothesis 1a. However, it should be noted that the manipulation of candidate competence was not successful, meaning that it was exposure to salient competence‐related traits, rather than perceived high competence, that influenced such preferences. In contrast, Study 2 yielded non‐robust evidence that a candidate's high warmth (vs. neutral traits) increased preferences for the candidate slightly more among voters low in RWA than those high in RWA, as this interactive effect of candidate warmth and RWA was only significant on the feeling thermometer (and marginally significant on overall evaluations and the likelihood of voting). It thus provided limited support for Hypothesis 2a. Additionally, our results did not support hypotheses regarding the moderating role of SDO in the relationships between candidate traits and voting preferences. Nor did the results support the predicted moderated mediations—that is, the indirect effects of candidate competence on voting preferences through the candidate's perceived ability to clean up danger and perceived ability to win competitions, or the indirect effect of candidate warmth on voting preferences through the candidate's perceived caring about people, being moderated by RWA and SDO.

### Implications

The findings from Study 1 support the prediction that high competence (vs. neutral traits) increases preferences for the candidate particularly among voters high in RWA. As prior research suggests, individuals high in RWA perceive the world as a dangerous place and are sensitive to external threats (Cohrs & Asbrock, 2009; Dallago et al., 2012). Competence, as a signal of intelligence, capability and problem‐solving skills, appears to fulfil high authoritarians' psychological need for security. Moreover, Study 2 provided some weak indication that high warmth (vs. neutral traits) could increase preferences for the candidate (particularly on the feeling thermometer) among voters low in RWA, but the results were not robust. Warmth, which reflects traits such as sociability, morality and trustworthiness, may resonate with the psychological needs of voters who perceive the world as safe, as they prioritize reciprocity and collective well‐being over the pursuit of security (Cottrell & Neuberg, 2005; Eisenbruch & Krasnow, 2022).

Contrary to our hypotheses, SDO had very limited moderating effects, particularly regarding the relationship between candidate competence and voting preferences. One potential explanation is that our operationalization of competence primarily reflected ability (i.e. intelligence and skills) and assertiveness (i.e. ambition and self‐confidence), rather than dominance. While competence and dominance perceptions are often correlated (Chen et al., 2014), previous research has suggested that perceptions of these traits can have distinct—and sometimes opposing—effects on voting preferences under certain conditions (e.g. Long et al., 2024; Sprong et al., 2019). However, since SDO is particularly linked to a preference for hierarchical dominance (Ho et al., 2015), its influence on voters' preferences for competent political candidates might have been muted when participants did not necessarily perceive competence as a marker of dominance. Another possible reason is the low relevance of SDO in non‐competitive contexts. That is, the current studies may not have adequately activated intergroup competition or hierarchical concerns, which are central to SDO (Does & Mentovich, 2016; Ho et al., 2015; Hoyt & Simon, 2016). Instead, voters evaluated candidates in a relatively neutral context that did not highlight zero‐sum competition (e.g. intergroup conflict or resource scarcity). In the absence of these situational triggers, the role of SDO in shaping the relationships between candidate traits and voting preferences may be minimal.

Moreover, we did not find evidence supporting our proposed moderated mediations. The non‐significant findings regarding moderated mediations suggest that individuals may tend to base their voting decisions on simpler and more heuristic cues (Lau & Redlawsk, 2001). While people often believe that rational and deliberative considerations are essential for making voting decisions, previous research indicates that rapid, unreflective inferences of candidate traits play a crucial role in shaping voting choices (Todorov et al., 2005). This tendency is particularly evident in low‐information contexts, such as in our study, where participants lacked comprehensive knowledge about the candidates presented, including their party affiliations, policy positions, and electoral records (Johns & Shephard, 2007). Additionally, the nature of online experiments may have further limited participants' focus on the functional implications of candidate traits (e.g. problem‐solving or caring for others). Unlike real elections, our online experiments carried significantly less importance and demanded less cognitive effort, which may have led participants to focus on general impressions of competence and warmth rather than considering what these traits meant in a more nuanced or practical context.

In addition to the main findings discussed above, the supplementary results regarding the main effects of candidate competence/warmth and RWA/SDO presented in Appendix S1 are worth mentioning. First, as shown in Tables S1.2 and S1.4, individuals preferred a highly warm candidate over a neutral one on all voting preference measures, whereas no significant difference in voting preferences was observed between a highly competent candidate and a neutral one. These findings appear to align with prior research on the primacy of warmth (over competence) in social judgment (Fiske, 2018; Laustsen & Bor, 2017). However, the asymmetrical influence of candidate warmth and competence may also be attributed to an unsuccessful manipulation of candidate competence, which could have led to the absence of a significant manipulation effect on voting preference.

Interestingly, RWA in both studies, and SDO in Study 1, significantly predicted all voting preference measures: the higher a voter's RWA or SDO score, the more favourable their evaluations of politicians in general, the more positive their feelings towards them, and the greater their likelihood of voting for them. This pattern may reflect the psychological foundations of these ideologies: individuals high in RWA tend to respect authority, while those high in SDO prefer group‐based dominance and hierarchy (Altemeyer, 1981; Mallinas et al., 2020; Sidanius & Pratto, 1999). Given that politicians are often perceived as authority figures or symbols of dominance, they may be especially appealing to individuals high in RWA and/or SDO. These findings may also be understood through the lens of system justification theory (Jost et al., 2004; Jost & Banaji, 1994): individuals high in RWA and/or SDO are more likely to legitimize political systems; they thus have more favourable views of politicians who are often seen as representatives of existing power structures.

### Limitations

A major limitation of the current studies concerns the manipulation of candidate competence. According to our manipulation check, there was no significant difference in perceived candidate competence between the high‐competence and neutral conditions. As the analysis suggested, a ‘neutral’ candidate was perceived as already highly competent (*M* = 5.67, *SD* = 0.85, on a 1–7 scale) by participants. This is perhaps unsurprising, as an incompetent individual is unlikely to be considered a viable candidate for a legislative seat. Consequently, even a ‘neutral’ (but viable) candidate can be naturally perceived as competent. Notably, previous research has faced a similar challenge when attempting to present a less competent political candidate in a control condition (e.g. Laustsen & Bor, 2017). Future studies examining candidate traits should aim to develop a well‐validated approach for manipulating candidate competence.

Another limitation lies in the operationalization of competence and warmth. While we focused on higher‐order constructs, emerging literature suggests that the “Big Two” dimensions consist of distinct subcomponents (e.g. ability and assertiveness for competence, and sociability and morality for warmth; Abele et al., 2016; Brambilla & Leach, 2014). However, as examining these four subcomponents was beyond the scope of the current research and would have added considerable complexity to the study design, we did not investigate the subcomponents of competence and warmth separately. Additionally, a major challenge in studying these subcomponents is their potential overlap (Abele, 2022)—between ability and assertiveness, or between sociability and morality—which makes it difficult to disentangle their ‘purely’ individual effects. Future attempts should be made to address these issues, exploring whether a certain subcomponent of competence and warmth is most influential in shaping voting preferences and whether this effect varies as a function of individual differences, such as RWA or SDO.

Moreover, although we focused on RWA and SDO, it is important to acknowledge that other ideological variables, such as populism, egalitarianism, and political cynicism (Donovan, 2021; Sheehy‐Skeffington & Thomsen, 2020; Van Hiel et al., 2022), may also interact with candidate traits to influence voting preferences. For example, research has shown that populist voters are particularly drawn to political candidates with high levels of dark traits (Nai, 2022). Future research may explore these additional variables to provide a more comprehensive understanding of how ideological beliefs shape preferences for candidates with different personal traits.

Finally, as our studies were conducted within the British context, the generalizability of our findings to other socio‐cultural settings remains uncertain. Cultural factors, such as individualism and cultural tightness, have been shown to influence voters' preferences for leaders with specific traits (Chen et al., 2012, 2024). Therefore, cross‐cultural studies are needed to figure out whether (and if so, how) cultural differences affect the ways in which RWA and SDO shape preferences for competent and warm candidates.

### Conclusion

The current research demonstrates that a political candidate's competence is particularly valued by voters high in RWA, while only non‐robust evidence indicates that candidate warmth is especially favoured by those low in RWA. In highlighting this asymmetry, the present studies advance our understanding of the interplay between candidate traits and voters' ideological beliefs, contributing to both social psychology and political science. However, the limited role of SDO and non‐significant moderated mediations underscore the complexity of voter decision‐making processes and the need for further investigation. Future research should address the identified limitations and explore additional variables and contexts to build a more comprehensive understanding of how political candidates' personal characteristics shape voting choices. Such efforts would inform politicians of strategies to effectively communicate their strengths during election campaigns.

## AUTHOR CONTRIBUTIONS

**Feiteng Long:** Conceptualization; methodology; software; data curation; investigation; validation; formal analysis; supervision; resources; project administration; visualization; writing – original draft; writing – review and editing; funding acquisition. **Zi Ye:** Funding acquisition; conceptualization; writing – review and editing.

## FUNDING INFORMATION

This work was supported by the Social, Economic and Organisational Psychology Unit at Leiden University.

## Supporting information


Appendices S1–S4

## Data Availability

The data that support the findings of this study are openly available in OSF at https://doi.org/10.17605/OSF.IO/X3DP7.

## Footnotes

^1^
The Stage 1 registered report stated, ‘The composite score is obtained by converting overall evaluations to a 0–100 scale and averaging the converted overall evaluation, feeling thermometer, and likelihood of voting measures’. However, we realized that the overall evaluation measure was already a composite of three items and, therefore, might not be highly comparable to the other two outcome variables. Moreover, the proposed rescaling would not have addressed potential differences in variability or distribution between scales. Hence, following the most common practice (Howell, 2013), we decided to deviate from the preregistration, obtaining a composite of voting preference by averaging the *z*‐scores of the overall evaluation, feeling thermometer, and likelihood of voting measures. This approach ensures that all measures contribute equally to the composite.
