# Supplementary material for: Who values competent minds and who likes warm hearts? The role of right‐wing authoritarianism and social dominance orientation in shaping voter preferences for political candidates
Source: Br J Soc Psychol. 2025 Jul 6;64(3):e70004. doi: 10.1111/bjso.70004 (PMC12230378; doi:10.1111/bjso.70004)
Supplement: Supplementary file 1 — Appendices S1–S4 [file BJSO-64-0-s001.docx]

**Appendix S1. Descriptive Statistics, Bivariate Correlations, and *t*-Tests**

**Table S1.1**

*Means, Standard Deviations, and Bivariate Correlations Between Measures in Study 1*

|  | *M* | *SD* | 1 | 2 | 3 | 4 | 5 | 6 | 7 | 8 | 9 |
| --- | --- | --- | --- | --- | --- | --- | --- | --- | --- | --- | --- |
| 1. Perceived candidate competence | 5.72 | 0.80 | — |  |  |  |  |  |  |  |  |
| 2. Overall evaluations | 5.09 | 1.13 | .56*** | — |  |  |  |  |  |  |  |
| 3. Feeling thermometer | 63.80 | 17.69 | .51*** | .84*** | — |  |  |  |  |  |  |
| 4. Likelihood of voting | 58.99 | 20.36 | .45*** | .79*** | .88*** | — |  |  |  |  |  |
| 5. Voting preference (composite) | –0.00 | 0.94 | .54*** | .93*** | .96*** | .94*** | — |  |  |  |  |
| 6. Perceived ability to clean up danger | 4.55 | 1.16 | .42*** | .55*** | .57*** | .55*** | .59*** | — |  |  |  |
| 7. Perceived ability to win competitions | 5.07 | 0.93 | .48*** | .50*** | .46*** | .43*** | .49*** | .55*** | — |  |  |
| 8. Perceived caring about people | 4.65 | 1.26 | .46*** | .71*** | .75*** | .70*** | .76*** | .59*** | .48*** | — |  |
| 9. RWA | 3.31 | 1.12 | .08 | .27*** | .28*** | .28*** | .29*** | .11* | .11* | .27*** | — |
| 10. SDO | 2.69 | 1.17 | .05 | .18*** | .19*** | .20*** | .20*** | –.02 | .01 | .14** | .54*** |

*Note*. * *p* < .050; ** *p* < .010; *** *p* < .001.

**Table S1.2**

*Means and Standard Deviations for Outcome Variables and Mediators Between Experimental Conditions, Along with Independent Samples t-Test Results in Study 1*

| Outcome variable/ mediator | Between-condition *M* (*SD*) | | *t*-test |
| --- | --- | --- | --- |
|  | High competence | Neutral |  |
| Overall evaluations | 5.14 (1.14) | 5.05 (1.11) | *t*(435) = 0.86, *p* = .390, *d* = 0.08 |
| Feeling thermometer | 64.31 (17.45) | 63.30 (17.96) | *t*(435) = 0.59, *p* = .554, *d* = 0.06 |
| Likelihood of voting | 59.50 (20.37) | 58.49 (20.39) | *t*(435) = 0.52, *p* = .606, *d* = 0.05 |
| Voting preference (composite) | 0.03 (0.94) | –0.03 (0.95) | *t*(435) = 0.70, *p* = .487, *d* = 0.07 |
| Perceived ability to clean up danger | 4.51 (1.09) | 4.59 (1.23) | *t*(435) = 0.78, *p* = .434, *d* = 0.07 |
| Perceived ability to win competitions | 5.19 (0.93) | 4.95 (0.92) | *t*(435) = 2.73, *p* = .007, *d* = 0.26 |
| Perceived caring about people | 4.55 (1.29) | 4.75 (1.21) | *t*(435) = 1.68, *p* = .093, *d* = 0.16 |

**Table S1.3**

*Means, Standard Deviations, and Bivariate Correlations Between Measures in Study 2*

|  | *M* | *SD* | 1 | 2 | 3 | 4 | 5 | 6 | 7 | 8 | 9 |
| --- | --- | --- | --- | --- | --- | --- | --- | --- | --- | --- | --- |
| 1. Perceived candidate warmth | 5.16 | 1.08 | — |  |  |  |  |  |  |  |  |
| 2. Overall evaluations | 5.10 | 1.18 | .71*** | — |  |  |  |  |  |  |  |
| 3. Feeling thermometer | 67.09 | 19.84 | .71*** | .85*** | — |  |  |  |  |  |  |
| 4. Likelihood of voting | 61.53 | 23.13 | .63*** | .80*** | .87*** | — |  |  |  |  |  |
| 5. Voting preference (composite) | –0.00 | 0.95 | .72*** | .94*** | .96*** | .94*** | — |  |  |  |  |
| 6. Perceived ability to clean up danger | 4.39 | 1.25 | .44*** | .58*** | .53*** | .57*** | .59*** | — |  |  |  |
| 7. Perceived ability to win competitions | 4.46 | 1.02 | .23*** | .41*** | .27*** | .31*** | .35*** | .52*** | — |  |  |
| 8. Perceived caring about people | 5.16 | 1.37 | .73*** | .71*** | .74*** | .70*** | .76*** | .53*** | .19*** | — |  |
| 9. RWA | 3.45 | 1.02 | .16*** | .17*** | .17*** | .22*** | .20*** | .20*** | .16*** | .20*** | — |
| 10. SDO | 2.66 | 1.15 | –.04 | –.00 | .04 | .04 | .03 | –.05 | –.09 | .00 | .44*** |

*Note*. *** *p* < .001.

**Table S1.4**

*Means and Standard Deviations for Outcome Variables and Mediators Between Experimental Conditions, Along with Independent Samples t-Test Results in Study 2*

| Outcome variable/ mediator | Between-condition *M* (*SD*) | | *t*-test |
| --- | --- | --- | --- |
|  | High warmth | Neutral |  |
| Overall evaluations | 5.40 (1.07) | 4.80 (1.22) | *t*(442) = 5.58, *p* < .001, *d* = 0.53 |
| Feeling thermometer | 73.93 (17.29) | 60.11 (19.86) | *t*(442) = 7.82, *p* < .001, *d* = 0.74 |
| Likelihood of voting | 67.68 (21.38) | 55.27 (23.21) | *t*(442) = 5.86, *p* < .001, *d* = 0.56 |
| Voting preference (composite) | 0.29 (0.83) | –0.29 (0.96) | *t*(442) = 6.81, *p* < .001, *d* = 0.65 |
| Perceived ability to clean up danger | 4.37 (1.24) | 4.42 (1.26) | *t*(442) = 0.41, *p* = .679, *d* = 0.04 |
| Perceived ability to win competitions | 4.20 (1.01) | 4.73 (0.96) | *t*(442) = 5.74, *p* < .001, *d* = 0.54 |
| Perceived caring about people | 5.75 (1.15) | 4.55 (1.31) | *t*(442) = 10.29, *p* < .001, *d* = 0.98 |

**Appendix S2. Factor Analysis**

**Table S2.1**

*Principal Component Analysis for Perceived Ability to Clean Up Danger (Danger), Perceived Ability to Win Competitions (Competition), and Perceived Caring about People (Caring) Scales in Study 1*

|  | Component | | |  |
| --- | --- | --- | --- | --- |
|  | 1 | 2 | 3 | Uniqueness |
| Danger1 | .03 | **.84** | –.02 | .28 |
| Danger2 | –.03 | **.94** | .00 | .14 |
| Danger3 | .03 | **.86** | .04 | .19 |
| Competition1 | .07 | .12 | **.57** | .54 |
| Competition2 | –.03 | –.09 | **.79** | .46 |
| Competition3 | .07 | .19 | **.66** | .32 |
| Caring1 | **.94** | .03 | –.07 | .14 |
| Caring2 | **.89** | –.06 | .09 | .18 |
| Caring3 | **.88** | .03 | .00 | .20 |

*Note.* “Minimum residual” extraction method was used in combination with a “oblimin” rotation.

**Table S2.1**

*Principal Component Analysis for Perceived Ability to Clean Up Danger (Danger), Perceived Ability to Win Competitions (Competition), and Perceived Caring about People (Caring) Scales in Study 2*

|  | Component | | |  |
| --- | --- | --- | --- | --- |
|  | 1 | 2 | 3 | Uniqueness |
| Danger1 | .09 | **.84** | –.02 | .21 |
| Danger2 | –.04 | **.96** | –.01 | .13 |
| Danger3 | –.01 | **.89** | .05 | .17 |
| Competition1 | .05 | .02 | **.76** | .39 |
| Competition2 | –.09 | –.09 | **.76** | .50 |
| Competition3 | .07 | .18 | **.65** | .38 |
| Caring1 | **.90** | .02 | –.04 | .18 |
| Caring2 | **.89** | .02 | .01 | .17 |
| Caring3 | **.95** | –.04 | .03 | .12 |

*Note.* “Minimum residual” extraction method was used in combination with a “oblimin” rotation.

**Appendix S3. Exploratory Analyses with Perceived Candidate Competence/Warmth**

As we found that the manipulation did not change participants’ perceptions of candidate competence in Study 1, we conducted parallel analyses with perceived candidate competence rather than the manipulated candidate competence. For an exploratory purpose, we conducted the same analyses for Study 2.

**Study 1**

First, we regressed each of the four voting preference measures (i.e., overall evaluations, the feeling thermometer, the likelihood of voting, and the voting preference composite) on perceived candidate competence, RWA/SDO, and their interaction. RWA and SDO were included in separate models in order to fully account for their influences without controlling for each other. As in the main text, we focused on the moderating roles of RWA and SDO in the relationship between perceived candidate competence and each outcome variable.

As shown in Table S3.1, regarding the moderating role of RWA, the interactive effect of perceived candidate competence and RWA was significant only on overall evaluations but not on other outcome variables. Decomposition of the significant interaction suggested that perceived candidate competence positively predicted overall evaluations among individuals both high and low in RWA, yet the effect was larger among those high in RWA (high RWA: *b* = 0.87, *SE* = 0.07, *p* < .001; low RWA: *b* = 0.65, *SE* = 0.08, *p* < .001). The interaction between perceived candidate competence and SDO, however, did not influence any of the four outcome variables, as Table S3.1 suggests.

We then tested moderated mediation using PROCESS Model 15 (Hayes, 2013), with perceived candidate competence as the independent variable, RWA/SDO as the moderator, and each of the four voting preference measures as the dependent variable. In addition, perceived ability to clean up danger, perceived ability to win competitions, and perceived caring about people were specified as parallel mediators. As shown in Table S3.2, the indirect effects of perceived candidate competence on outcome variables through perceived ability to clean up danger were not moderated by RWA, and the indirect effects of perceived candidate competence on outcome variables through perceived ability to win competitions were not moderated by SDO.

**Study 2**

We conducted the same analyses for Study 2 with perceived candidate warmth. As shown in Table S3.3, neither the interaction between perceived candidate warmth and RWA nor that between perceived candidate warmth and SDO significantly influenced any of the four outcome variables. In terms of moderated mediation shown in Table S3.4, again, the indirect effects of perceived candidate warmth on outcome variables through perceived ability to clean up danger were not moderated by RWA, and the indirect effects of perceived candidate warmth on outcome variables through perceived ability to win competitions were not moderated by SDO.

**Table S3.1**

*The Interactive Effect of the Perceived Candidate Competence and RWA/SDO on Outcome Variables in Study 1*

| Predictor | Outcome variable | | | | | | |
| --- | --- | --- | --- | --- | --- | --- | --- |
|  | Overall evaluations | | Feeling thermometer | | Likelihood of voting | | Voting preference  (composite) |
| *Moderator: RWA* |  |  | |  | |  | |
| Perceived competence | 0.42 (0.17), *p* = .013 | | 8.99 (2.77), *p* = .001 | | 8.25 (3.31), *p* = .013 | | 0.43 (0.14), *p* = .003 |
| RWA | –0.36 (0.28), *p* = .198 | | 0.56 (4.50), *p* = .901 | | –0.01 (5.39), *p* = .999 | | –0.09 (0.23), *p* = .685 |
| Perceived competence × RWA | 0.10 (0.05), *p* = .034 | | 0.56 (0.78), *p* = .468 | | 0.79 (0.93), *p* = .395 | | 0.05 (0.04), *p* = .186 |
| *Moderator: SDO* |  |  | |  | |  | |
| Perceived competence | 0.74 (0.14), *p* < .001 | | 12.66 (2.28), *p* < .001 | | 12.72 (2.73), *p* < .001 | | 0.67 (0.12), *p* < .001 |
| SDO | 0.06 (0.28), *p* = .825 | | 5.68 (4.52), *p* = .209 | | 6.37 (5.39), *p* = .238 | | 0.23 (0.24), *p* = .331 |
| Perceived competence × SDO | 0.02 (0.05), *p* = .744 | | –0.56 (0.79), *p* = .476 | | –0.56 (0.94), *p* = .549 | | –0.02 (0.04), *p* = .712 |

*Note*. Entries are unstandardized regression coefficients with standard errors in parentheses, followed by *p*-values.

**Table S3.2**

*Indirect Effects of Perceived Candidate Competence Moderated by RWA/SDO in Study 1*

| Mediator | Moderator: RWA | | | Moderator: SDO | | |
| --- | --- | --- | --- | --- | --- | --- |
|  | Estimate | *SE* | 95% CI | Estimate | *SE* | 95% CI |
| *Indirect effect of candidate competence on overall evaluations* | | | | | | |
| Perceived ability to clean up danger | 0.03 | 0.03 | [–0.02, 0.09] | –0.05 | 0.02 | [–0.09, –0.00] |
| Perceived ability to win competitions | 0.01 | 0.03 | [–0.05, 0.07] | 0.02 | 0.03 | [–0.04, 0.08] |
| Perceived caring about people | –0.03 | 0.03 | [–0.10, 0.02] | –0.01 | 0.03 | [–0.06, 0.05] |
| *Indirect effect of candidate competence on feeling thermometer* | | | | | | |
| Perceived ability to clean up danger | 0.52 | 0.40 | [–0.25, 1.32] | –0.57 | 0.40 | [–1.40, 0.18] |
| Perceived ability to win competitions | 0.22 | 0.46 | [–0.68, 1.18] | 0.59 | 0.45 | [–0.26, 1.53] |
| Perceived caring about people | –0.56 | 0.46 | [–1.46, 0.35] | –0.09 | 0.45 | [–0.95, 0.81] |
| *Indirect effect of candidate competence on likelihood of voting* | | | | | | |
| Perceived ability to clean up danger | 0.46 | 0.57 | [–0.75, 1.53] | –0.73 | 0.57 | [–1.91, 0.35] |
| Perceived ability to win competitions | –0.10 | 0.60 | [–1.25, 1.16] | –0.10 | 0.58 | [–1.27, 1.00] |
| Perceived caring about people | –0.26 | 0.68 | [–1.54, 1.15] | 0.15 | 0.66 | [–1.10, 1.49] |
| *Indirect effect of candidate competence on voting preference (composite)* | | | | | | |
| Perceived ability to clean up danger | 0.03 | 0.02 | [–0.02, 0.07] | –0.04 | 0.02 | [–0.08, 0.00] |
| Perceived ability to win competitions | 0.01 | 0.02 | [–0.04, 0.06] | 0.01 | 0.02 | [–0.03, 0.06] |
| Perceived caring about people | –0.02 | 0.03 | [–0.08, 0.03] | –0.00 | 0.02 | [–0.05, 0.05] |

*Note*. Confidence intervals (CIs) were obtained with 5,000 bootstrap replicates.

**Table S3.3**

*The Interactive Effect of the Perceived Candidate Warmth and RWA/SDO on Outcome Variables in Study 1*

| Predictor | Outcome variable | | | | | | |
| --- | --- | --- | --- | --- | --- | --- | --- |
|  | Overall evaluations | | Feeling thermometer | | Likelihood of voting | | Voting preference  (composite) |
| *Moderator: RWA* |  |  | |  | |  | |
| Perceived warmth | 0.87 (0.12), *p* < .001 | | 16.02 (2.04), *p* < .001 | | 13.18 (2.63), *p* < .001 | | 0.70 (0.10), *p* < .001 |
| RWA | 0.22 (0.18), *p* = .225 | | 5.98 (3.08), *p* = .053 | | 3.05 (3.96), *p* = .441 | | 0.21 (0.14), *p* = .151 |
| Perceived warmth × RWA | –0.03 (0.03), *p* = .395 | | –0.92 (0.57), *p* = .111 | | –0.05 (0.74), *p* = .949 | | –0.02 (0.03), *p* = .366 |
| *Moderator: SDO* |  |  | |  | |  | |
| Perceived warmth | 0.93 (0.09), *p* < .001 | | 13.35 (1.45), *p* < .001 | | 13.75 (1.87), *p* < .001 | | 0.68 (0.07), *p* < .001 |
| SDO | 0.32 (0.16), *p* = .042 | | 1.59 (2.61), *p* = .543 | | 1.73 (3.39), *p* = .609 | | 0.14 (0.12), *p* = .250 |
| Perceived warmth × SDO | –0.06 (0.03), *p* = .059 | | –0.08 (0.50), *p* = .873 | | –0.09 (0.64), *p* = .885 | | –0.02 (0.02), *p* = .427 |

*Note*. Entries are unstandardized regression coefficients with standard errors in parentheses, followed by *p*-values.

**Table S3.4**

*Indirect Effects of Perceived Candidate Warmth Moderated by RWA/SDO in Study 1*

| Mediator | Moderator: RWA | | | Moderator: SDO | | |
| --- | --- | --- | --- | --- | --- | --- |
|  | Estimate | *SE* | 95% CI | Estimate | *SE* | 95% CI |
| *Indirect effect of candidate warmth on overall evaluations* | | | | | | |
| Perceived ability to clean up danger | 0.02 | 0.02 | [–0.02, 0.05] | 0.00 | 0.02 | [–0.03, 0.04] |
| Perceived ability to win competitions | –0.00 | 0.01 | [–0.02, 0.02] | –0.00 | 0.01 | [–0.02, 0.01] |
| Perceived caring about people | –0.02 | 0.04 | [–0.09, 0.04] | 0.01 | 0.04 | [–0.06, 0.08] |
| *Indirect effect of candidate warmth on feeling thermometer* | | | | | | |
| Perceived ability to clean up danger | 0.42 | 0.30 | [–0.14, 1.04] | –0.10 | 0.28 | [–0.61, 0.47] |
| Perceived ability to win competitions | –0.06 | 0.15 | [–0.38, 0.24] | 0.12 | 0.14 | [–0.14, 0.41] |
| Perceived caring about people | 0.23 | 0.71 | [–1.28, 1.55] | 0.15 | 0.64 | [–1.18, 1.39] |
| *Indirect effect of candidate warmth on likelihood of voting* | | | | | | |
| Perceived ability to clean up danger | 0.48 | 0.39 | [–0.29, 1.25] | –0.11 | 0.33 | [–0.72, 0.55] |
| Perceived ability to win competitions | –0.19 | 0.19 | [–0.60, 0.17] | 0.16 | 0.17 | [–0.17, 0.53] |
| Perceived caring about people | –0.60 | 0.90 | [–2.28, 1.26] | –0.56 | 0.76 | [–2.10, 0.86] |
| *Indirect effect of candidate warmth on voting preference (composite)* | | | | | | |
| Perceived ability to clean up danger | 0.02 | 0.01 | [–0.01, 0.05] | –0.00 | 0.01 | [–0.03, 0.02] |
| Perceived ability to win competitions | –0.00 | 0.01 | [–0.02, 0.01] | 0.00 | 0.01 | [–0.01, 0.02] |
| Perceived caring about people | –0.01 | 0.03 | [–0.07, 0.05] | –0.00 | 0.03 | [–0.06, 0.05] |

*Note*. Confidence intervals (CIs) were obtained with 5,000 bootstrap replicates.

**Appendix S4. Exploratory Mediation Analyses on the Indirect Effects of Candidate Competence/Warmth on Voting Preferences**

To explore whether the manipulation of candidate traits indirectly influenced voting preference measures through perceived ability to clean up danger, perceived ability to win competitions, and perceived caring about people, we conducted additional mediation analyses using PROCESS Model 4 (Hayes, 2013).

**Study 1**

As shown in Table S4.1, candidate competence did not have robust indirect effects on outcome variables through perceived ability to clean up danger, perceived ability to win competitions, or perceived caring about people. The only significant indirect effect was on overall evaluations, mediated by perceived ability to win competitions.

**Study 2**

Results in Table S4.2 demonstrate that candidate warmth indirectly influenced all outcome variables through both perceived ability to win competitions and perceived caring about people. More specifically, highly warm (vs. neutral) traits indirectly reduced voting preference for the candidate by lowering perceptions of the candidate’s ability to win competitions, while simultaneously increasing voting preference by enhancing perceptions that the candidate would care about people.

**Table S4.1**

*Indirect Effects of Candidate Competence in Study 1*

| Mediator | Estimate | *SE* | 95% CI |
| --- | --- | --- | --- |
| *DV: overall evaluations* | | | |
| Perceived ability to clean up danger | –0.01 | 0.02 | [–0.04, 0.02] |
| Perceived ability to win competitions | 0.04 | 0.02 | [0.01, 0.10] |
| Perceived caring about people | –0.10 | 0.06 | [–0.23, 0.02] |
| *DV: feeling thermometer* | | | |
| Perceived ability to clean up danger | –0.23 | 0.30 | [–0.83, 0.38] |
| Perceived ability to win competitions | 0.25 | 0.23 | [–0.14, 0.80] |
| Perceived caring about people | –1.78 | 1.06 | [–3.85, 0.31] |
| *DV: likelihood of voting* | | | |
| Perceived ability to clean up danger | –0.28 | 0.38 | [–1.08, 0.46] |
| Perceived ability to win competitions | 0.24 | 0.28 | [–0.28, 0.87] |
| Perceived caring about people | –1.88 | 1.13 | [–4.17, 0.31] |
| *DV: voting preference (composite)* | | | |
| Perceived ability to clean up danger | –0.01 | 0.02 | [–0.04, 0.02] |
| Perceived ability to win competitions | 0.02 | 0.01 | [–0.00, 0.05] |
| Perceived caring about people | –0.09 | 0.06 | [–0.21, 0.02] |

*Note.* Confidence intervals (CIs) were obtained with 10,000 bootstrap replicates.

**Table S4.2**

*Indirect Effects of Candidate Warmth in Study 2*

| Mediator | Estimate | *SE* | 95% CI |
| --- | --- | --- | --- |
| *DV: overall evaluations* | | | |
| Perceived ability to clean up danger | –0.01 | 0.02 | [–0.04, 0.03] |
| Perceived ability to win competitions | –0.16 | 0.04 | [–0.24, –0.09] |
| Perceived caring about people | 0.57 | 0.08 | [0.42, 0.72] |
| *DV: feeling thermometer* | | | |
| Perceived ability to clean up danger | –0.13 | 0.34 | [–0.81, 0.56] |
| Perceived ability to win competitions | –1.13 | 0.50 | [–2.18, –0.23] |
| Perceived caring about people | 9.94 | 1.35 | [7.35, 12.70] |
| *DV: likelihood of voting* | | | |
| Perceived ability to clean up danger | –0.21 | 0.51 | [–1.22, 0.80] |
| Perceived ability to win competitions | –1.43 | 0.54 | [–2.55, –0.43] |
| Perceived caring about people | 10.70 | 1.47 | [7.93, 13.74] |
| *DV: voting preference (composite)* | | | |
| Perceived ability to clean up danger | –0.01 | 0.02 | [–0.04, 0.03] |
| Perceived ability to win competitions | –0.08 | 0.02 | [–0.13, –0.04] |
| Perceived caring about people | 0.48 | 0.06 | [0.36, 0.61] |

*Note.* Confidence intervals (CIs) were obtained with 10,000 bootstrap replicates.
